# A Descriptive Correlational Study to Evaluate Three Measures of Assessing Upper Extremity Function in Individuals with Multiple Sclerosis

**DOI:** 10.1155/2021/5588335

**Published:** 2021-06-26

**Authors:** Aman Saini, Audrey Zucker-Levin, Benjamin McMillan, Pawan Kumar, Sarah Donkers, Michael C. Levin

**Affiliations:** ^1^Office of the Saskatchewan MS Clinical Research Chair, College of Medicine, University of Saskatchewan, Saskatchewan, Canada; ^2^School of Rehabilitation Science, College of Medicine, University of Saskatchewan, Saskatchewan, Canada; ^3^College of Kinesiology, University of Saskatchewan, Saskatchewan, Canada; ^4^Department of Medicine, Neurology Division, College of Medicine, University of Saskatchewan, Saskatchewan, Canada; ^5^Department of Anatomy, Physiology and Pharmacology, College of Medicine, University of Saskatchewan, Saskatchewan, Canada

## Abstract

**Background:**

Activities of daily living and quality of life (QOL) are hindered by upper extremity (UE) impairments experienced by individuals with multiple sclerosis (iMS). The Nine-Hole Peg Test (9-HPT) is most frequently used to measure UE function. However, it does not measure peoples' ability to perform routine tasks in daily life and may not be useful in iMS who cannot pick up the pegs utilized in the 9-HPT. Therefore, we evaluated three measures to explore a more comprehensive assessment of UE function: Upper Extremity Function Scale (UEFS), Action Research Arm Test (ARAT), and the 9-HPT. The objectives were to quantitatively assess the relationship between these measures of UE function, understand if the measures correlate with QOL as calculated by the MS Quality of Life-54 (MSQOL-54), and to determine differences in the measures based on employment status.

**Methods:**

112 (79 female) iMS were prospectively recruited for this descriptive correlational study. Inclusion criteria were as follows: confirmed diagnosis of MS or clinically isolated syndrome, age ≥ 18 years, and ability to self-consent. All statistical analyses including Spearman's correlation coefficient (*r*_*s*_) and Kruskal-Wallis tests were performed using SPSS.

**Results:**

A moderate correlation (*r*_*s*_ = −0.51; *p* < 0.001) was found between the ARAT and 9-HPT scores for the more impaired hand. Likewise, a moderate correlation was found between UEFS and the physical health composite scores (PHCSs) of MSQOL-54 (*r*_*s*_ = −0.59; *p* < 0.001). Finally, performances on ARAT, 9-HPT, and UEFS differed between the employed individuals and those on long-term disability (*p* = 0.007, *p* < 0.001, and *p* = 0.001).

**Conclusion:**

The UEFS moderately correlated with the QOL measure, and considering the UESF is a patient-reported outcome, it could be used to complement routinely captured measures of assessing UE function. Further study is warranted to determine which measure, or combination of measures, is more sensitive to changes in UE function over time.

## 1. Introduction

Upper extremity (UE) impairment, caused by a combination of motor and sensory deficits, hinders the ability of individuals with multiple sclerosis (iMS) to perform activities of daily living (ADL) and decreases their quality of life (QOL) [1]. UE impairment is widely reported in iMS affecting proximal and/or distal parts of the upper limbs and is associated with unemployment and negative economic impact [2]. Bertoni et al. studied unilateral and bilateral upper limb dysfunction in 105 iMS and found diminished dexterity, as measured by the Nine-Hole Peg Test (9-HPT), in 75% of their study population [3]. Presently, there are several standardized tools available for clinical assessment of hand dexterity in iMS including the 9-HPT, the box and block test (BBT), and the Jebsen-Taylor Hand Function Test (JTT) [4], with the 9-HPT most frequently used in clinical practice and research. These commonly used tests do not provide a complete assessment of UE function as each focuses on either proximal arm/hand movements or manual dexterity. The high rate of UE dysfunction in iMS merits careful assessment of the location and type of dysfunction, for example, hand versus shoulder or fine versus gross motor control and any combination therein. Identification of a comprehensive UE outcome measure that could systematically assess more complex and integrated UE function in iMS is needed [4, 5]. Clinicians and researchers require a tool that evaluates all aspects of UE function including manipulation of small and large objects, upper arm movements (reaching, lifting, and transport of objects), and both fine and gross movement components of manual dexterity in iMS, which are indispensable to perform activities of daily living. In a systematic review, Santisteban et al. performed a systematic literature review and found 48 different measures used to report UE function in people with stroke. Both the Action Research Arm Test (ARAT) and the 9-HPT were among the measures used most frequently [6]. The ARAT is found to be extremely useful as a comprehensive and reliable tool evaluating UE function in various studies with stroke patients evaluating UE function across a wide spectrum of impairments [7, 8]. In addition to physical performance tests, the past decade has seen an increase in the use of patient-reported outcome measures (PROMs) for evaluation in clinical settings with few reports on clinical correlation [9]. The Upper Extremity Function Scale (UEFS), which is a PROM, is more likely to detect significant changes as a result of treatment or progression in patients with a variety of UE dysfunctions than traditionally used clinical measures [10]. The multitude of tests available, the increased use of PROM for patient assessment, and the limitations in the 9-HPT bring to question if a more comprehensive measure of assessing UE function, such as the ARAT and UEFS, would better correlate to QOL in iMS. On the basis of these considerations, the objectives of the present study were as follows: (1) to quantitatively assess the relationship between measures of assessing UE function (UEFS, ARAT, and 9-HPT), (2) to understand if the performances on these three measures of assessing UE function correlate with QOL as measured by the Multiple Sclerosis (MS) Quality of Life-54 (MSQOL-54), and (3) to determine differences in the scores obtained from these measures of assessing UE function based on employment status. The primary goal of this study was to evaluate different means of assessing UE function: a PROM: (UEFS) and two physical assessments (ARAT and the 9-HPT) to determine which measure best correlates to QOL as measured by the MSQOL-54. The identified tool(s) can then be used to assess UE function in iMS, monitor for progression, and target appropriate intervention including physical and/or occupational therapy, with the overall objective of improving QOL in iMS.

## 2. Materials and Methods

### 2.1. Study Design and Population

A convenience sample of 112 iMS was prospectively recruited, consented, and evaluated for participation in this descriptive correlational study. Participants were evaluated in one of two locations: 44 participants were recruited at the Saskatchewan MS Connects Conference in November 2018 as a part of an interactive research clinic, and an additional 68 participants were recruited from the Saskatoon MS Clinic at Saskatoon City Hospital. One participant chose not to participate in the 9-HPT and ARAT but completed the UEFS and MSQOL-54; thus, only 111 participants were included in the analysis of the physical assessment measures. Individuals 18 years of age and older who have been physician diagnosed with MS or clinically isolated syndrome were included in this study. Those who were unable to consent for themselves and patients with medical conditions that preclude participation (previous surgery on the upper extremity, any other disorder that affected upper extremity function, serious acute/chronic comorbidities, or neurological disorders other than MS) were excluded from this study. Study data were collected and managed using Research Electronic Data Capture (REDCap), an electronic data capture tool hosted at the University of Saskatchewan. REDCap is a secure web-based platform that is specially designed to support data capture for research purposes [11, 12]. All consent documents, PROMs, and physical performance test data were collected on a tablet using REDCap version 9.3.7. When needed, an investigator assisted the participant with the tablet. Clinical demographic profiles (month and year of birth, sex, year of the first symptom, year of diagnosis, and MS phenotype) were collected from all participants at the time of data collection. Numbers of relapses, expanded disability status scale (EDSS), and employment status were collected from the clinic charts. Information on the current use of MS disease-modifying therapy (DMT) was collected from the participants and clinic charts. Ethics approval for this study was obtained from the University of Saskatchewan's Biomedical Research Ethics Board.

### 2.2. Tools

#### 2.2.1. Patient-Reported Outcome Measures (PROMs)

The Upper Extremity Functional Scale (UEFS) is an 8-item region-specific questionnaire developed to assess work-related upper extremity disorders. The UEFS is a valid, reliable, and responsive tool designed to measure the impact of upper extremity disorders on function in patients with a variety of diagnoses [10]. It is completed in less than 5 minutes. Participants reported their ability to perform 8 activities (sleeping, writing, opening jars, picking up small objects with fingers, driving a car for more than 30 minutes, carrying a milk jug from the refrigerator, opening a door, and washing dishes) by marking a line on a 0-10 visual analogue scale (VAS) with 0 indicating no problem and 10 indicating a major problem. The total score is calculated by adding VAS scores with possible scores ranging from 0 (best state) to 80 (worst state).

The Multiple Sclerosis Quality of Life-54 (MSQOL-54) is a multidimensional health-related quality of life self-report questionnaire with 11 domains that combine both generic and MS-specific items into a single instrument and can usually be completed with little or no assistance [13]. The MSQOL-54 demonstrates good internal consistency with high test-retest reliability and construct validity for assessing health-related quality of life in iMS [14, 15]. The 11 domains are physical function, pain, energy, emotional health, role limitations (physical/emotional), health-related perceptions, social function, health-related distress, sexual function, overall quality of life, and cognitive function. Composite scores are calculated for physical health (PHCS) and mental health (MHCS) with higher scores indicating better quality of life.

#### 2.2.2. Physical Performance Tests

The Nine-Hole Peg Test (9-HPT) is the most frequently used quantitative measure for upper extremity function, specifically hand dexterity, in MS. The 9-HPT has high interrater reliability, high test-retest reliability, and high discriminative validity [16]. The test is standardized with both hands (dominant and nondominant) tested twice by timing the participant as they place and then remove 9 pegs on a standardized pegboard. Each trial has a maximum 5-minute (300 second) time limit with 300 seconds recorded if the task could not be completed in the allotted time due to physical limitation. The mean time to complete the task, in seconds, is calculated for each hand [17] with lower scores indicating faster (better) performance. The faster-performing hand was identified as the “less impaired hand”; the other hand was identified as the “more impaired hand.” The average of all four trials (both hands were tested twice) was considered as the mean time for both hands.

The Action Research Arm Test (ARAT) is a standardized measure of arm and hand function which consists of 19 items organized in four different sections: grasp, grip, pinch, and gross movement [7]. ARAT was chosen among other upper limb functional measures because it allows a comprehensive evaluation of arm and hand function during the execution of tasks which are quite similar to activities of daily living and could be performed on subjects who are not able to pick up a peg/block. A trained investigator scores each item based on a 4-point ordinal scale, with 0 = unable to perform any part of the relevant task, 1 = able to perform the task partially (e.g. can only lift the relevant object), 2 = able to complete the task; but with abnormally long time/clumsiness/great difficulty, and 3 = able to perform task completely and normally. Participants are first asked to perform the most difficult task within a subscale (grasp/grip/pinch/gross movement). If the participant passes the first task adequately with normal movement, no more tasks in the subscale are administered and all items in the subscale are scored a 3. Likewise, if a participant scores a 0 on the first task within a subscale and scores a 0 on the second task, no more tasks in the subscale are administered and all tasks in the subscale are scored a 0. If the participant scores other than described, all tasks within a subscale are scored. The maximum score for ARAT is 57 for each arm, with a higher score indicating better performance.

### 2.3. Statistical Analyses

Descriptive and inferential statistics were utilized to establish a clinical-demographic profile and relationships between various measures used in this study. The demographic data of our study sample and scores obtained from study measures were expressed as mean ± standard deviation (SD). Spearman's rank correlation coefficient (*r*_*s*_) determined relationships among all PROMs and physical performance tests. Correlations (*r*_*s*_) between 0 and 2.9 (0 and -.29) were interpreted as negligible correlation, 0.3 and 0.49 (-0.3 and -0.49) as low positive (negative) correlation, 0.5 and 0.69 (-0.5 and -0.69) as moderate positive (negative) correlation, 0.7 and 0.89 (-0.7 and -0.89) as high positive (negative) correlation, and 0.9 and 1 (-0.9 and 1) as very high positive (negative) correlation [18, 19]. The Kruskal-Wallis test determined differences in the scores obtained from three measures of assessing UE function (9-HPT, ARAT, and UEFS) stratified by employment status. All statistical analyses were performed using SPSS version 25 with *α* = 0.05 for statistical significance.

## 3. Results

### 3.1. Patient Characteristics and Scores Obtained from Study Measures

112 iMS (79 female and 33 male mean age 50.3 ± 12.5 years; mean duration of MS 17.1 ± 14.1 years; 71 RRMS, 23 SPMS, 15 PPMS, and 3 CIS) were included in this study.  Table 1 shows the clinical-demographic profile of our study population. The median EDSS was 2.75 with 0.41 ± 0.6 (range = 0‐3) mean relapses per year. 36 iMS (32.1%) were employed whereas 32 iMS (28.6%) were on long-term disability. 63 iMS (56.3%) were taking MS DMTs.  Table 2 describes the mean scores obtained from the measures of assessing UE function and the MSQOL-54. The mean ARAT score (both hands) was 54.1 ± 6.4, the mean 9-HPT score (both hands) was 29.1 ± 23.2 seconds, and the mean UEFS score was 22.4 ± 17.1.

### 3.2. Correlations between Various Study Measures

**Figure 1:**
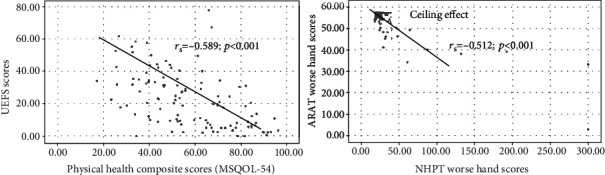
Correlation between study measures.

Figure 1illustrates the correlation between UEFS and PHCS, and ARAT more impaired hand and 9-HPT more impaired hand scores. A moderate negative correlation was found between the UEFS (higher score indicates worse function) and the PHCS (higher score indicates better QOL) (*r*_*s*_ = −0.59; *p* value <0.001). Likewise, a moderate negative correlation was also found between the ARAT (higher scores indicate better function) and 9-HPT scores (higher scores indicate worse function) for the more impaired hand (*r*_*s*_ = −0.51; *p* value <0.001).  Table 3 shows the correlations between various upper extremity functional scores (ARAT/9-HPT/UEFS) and MSQOL-54 scores. A low negative correlation was found between the 9-HPT both hands scores (higher score indicates worse function) and the PHCS (higher score indicates better QOL) (*r*_*s*_ = −0.36; *p* value <0.001). Also, a low positive correlation was found between the ARAT both hands score (higher scores indicate better function) and the PHCS (higher score indicates better QOL) (*r*_*s*_ = 0.33; *p* < 0.001).

### 3.3. Distribution of Upper Extremity Functional Scores according to Employment Status

The distributions of average ARAT, 9-HPT, and UEFS scores differed between employed individuals and those on long-term disability, with the employed individuals having better scores on the measures of assessing UE function than those on long-term disability (mean rank scores: ARAT employed = 69.13 and on long − term disability = 41.11, *p* = 0.007; 9-HPT employed = 33.44 and on long − term disability = 74.59, *p* < 0.001; UEFS employed = 39.36 and on long − term disability = 72.39, *p* = 0.001).

## 4. Discussion

UE dysfunction significantly contributes to disability in activities of daily living and could negatively impact QOL in iMS. A comprehensive assessment of UE function may provide additional information on the level of disability and might contribute to better planning of rehabilitation. Our results showed a statistically significant moderate correlation between the UEFS and the MSQOL-54 PHCS. A similar finding was observed by Paltamaa et al. who studied associations among measures of physical functioning and self-reported performance in mobility, domestic life, and self-care in ambulatory iMS. They found manual dexterity was a significant predictor of perceived difficulties in the performance of activities of daily living in ambulatory iMS [20]. Neurologic rating scales, such as the EDSS, are traditionally used to measure clinical disability in MS. However, EDSS has been criticized for lack of sensitivity specifically for evaluation of UE function, its high interrater variability, and its emphasis on ambulation [21, 22]. The Multiple Sclerosis Functional Composite (MSFC), consisting of three quantitative objective assessments to detect changes in ambulation, UE function, and cognition, was developed to address these limitations [23]. The 9-HPT, a component of MSFC, is now a frequently used measure to detect a change in UE function in iMS both in clinical practice and research [16]. However, the disadvantage of 9-HPT is its inability to detect proximal weakness, and it may not be useful in detecting UE impairment or progression of impairment in iMS who cannot pick up the pegs used in the 9-HPT.

Preservation of UE function in iMS is considered a potential treatment benefit. In individuals with restricted walking ability, maintaining UE function is of paramount importance as this could affect a person's ability to use walking aids [24]. The severity of UE impairment in iMS was suggested in a study in which 51% of the study sample (*n* = 285) reported at least moderate difficulty in hand function [25]. We found performance on the three UE function measures differed between employed iMS and those who were on long-term disability, with employed individuals had better mean rank scores on all three measures than those on long-term disability. This finding is in line with a study conducted by Marrie et al. who found an association of UE dysfunction with decreased odds of being employed (OR 0.97; 95% CI: 0.96, 0.98) and showed currently employed iMS had higher UE function scores than unemployed patients [2]. However, it is often difficult to ascertain and measure the variety of functional domains leading to UE impairments in iMS. Therefore, there remains a need for a measure of assessing UE function that could adequately capture UE impairments in individuals with greater levels of disability and measure peoples' ability to perform routine tasks in daily life. The ARAT, with its subscales for grasp, grip, pinch, and gross movements, could provide a more comprehensive functional assessment in iMS, and the UEFS might be valuable in providing the patient's perspective on the magnitude of UE dysfunction.

PROMs are increasingly being recommended for use as integral components in clinical trials [26]. Our analyses indicate UEFS (PROM) scores had weak correlations with performance on the ARAT and the 9-HPT. These findings align with those of Feys et al. who studied 43 iMS with upper limb dysfunction and found a poor to moderate correlation of upper extremity performance-based measures (TEMPA, Jebsen Hand Function Test, and 9-HPT) with an ADL self-questionnaire [27]. These poor correlations between self-reported and objective measures may be due to an individuals' ability to adapt to impairment. However, PROMs provide the patient's perspective and could complement objective assessments by identifying outcomes not routinely captured during clinical assessment. We found a statistically significant moderate correlation between UEFS and the physical health composite scores of MSQOL-54. Such outcome measures provide additional information on the daily life difficulties experienced by iMS, and improvement in these performance measures is being considered as the ultimate goal of any treatment or rehabilitative strategies.

A limitation of this study is that our convenience sample was skewed to iMS with mild disability (median EDSS = 2.75) with few limitations in arm/hand strength and gross movements. Thus, a future study should include more individuals with higher levels of disability as measured by the EDSS scores and progressive forms of MS. Our analysis of the scatter plot indicates that the ARAT had a ceiling effect (when a high proportion of study participants (>20%) have the highest possible score [28]). This is in line with a previous study conducted by Lamers et al. to determine the relationship between clinical tests in MS and real-life arm performance involving 30 iMS and 30 healthy controls. They also found a ceiling effect in the ARAT for the dominant arm [29]. Recently, Solaro et al. reported a floor and ceiling effect for the 9-HPT in iMS with mild (EDSS < 3) and severe (EDSS > 6) disease, respectively. They also found individuals with PPMS have more hand asymmetry as measured by the 9-HPT [30]. However, these preliminary findings require further investigation to draw any firm conclusions on floor and ceiling effects. Another limitation is that majority of our study participants were recruited from a single MS center, and therefore, caution should be taken with the generalization of our study results. We also do not have longitudinal data on the outcome measures which could have provided additional information. A further limitation of this study is that descriptors of disease activity in terms of relapse and/or active lesions on brain/spinal cord magnetic resonance imaging scans were not addressed in the eligibility criteria. Future studies could be designed to compare upper extremity function in patients with active disease versus nonactive disease.

The selection of a relevant measure of assessing UE function depends on the intended purpose of evaluation and severity of UE dysfunction. While the 9-HPT is an objective measure of assessing UE function, it does not measure peoples' ability to perform routine tasks in daily life, and it focuses on finger dexterity and may not be useful in iMS who cannot pick up the pegs utilized in the 9-HPT. Recently, a few studies [29, 31] have shown that although scores on objective measures of UE function are within normal range, iMS still report UE disability affecting their performance on activities of daily living. Therefore, it would be ideal to comprehensively evaluate UE function using both subjective and objective measures of assessing to better understand UE disability in iMS. Our results suggest that the performance on the UEFS moderately correlates with the QOL measure, and therefore, it could be instrumental in providing additional information on the difficulties experienced by iMS when performing specific UE tasks.

## 5. Conclusions

The performance on UEFS significantly correlated with the quality of life measure, and therefore, it could complement routinely captured measures of assessing UE function in iMS. Further study is warranted to determine which test, or combination of tests, is more sensitive to changes in UE function in iMS over time. Such measurements of UE function may provide additional information on disability accrual and could enhance the planning of rehabilitation programs targeted to improve the quality of life in iMS.

## Figures and Tables

**Table 1:** Clinical-demographic profile of our study sample.

Variable	Frequencies (%) *n* = 112
Sex	
Females	79 (70.5%)
Males	33 (29.5%)
Mean age (year)	50.3 ± 12.5
Mean age of onset (year)	33.1 ± 11.6
Mean duration (year)	17.1 ± 14.1
MS phenotype	
CIS	3 (2.7%)
RRMS	71 (63.4%)
SPMS	23 (20.5%)
PPMS	15 (13.4%)
Mean relapses per year (range)	0.41 ± 0.6 (0‐3)
Median EDSS	2.75
Employment status	
Employed	36 (32.1%)
Long-term disability	32 (28.6%)
Retired	14 (12.5%)
Unemployed	8 (7.1%)
Adjusted employment	2 (1.8%)
Self-employed	5 (4.5%)
Unpaid employment	2 (1.8%)
Unknown	13 (11.6%)
MS DMTs	
Alemtuzumab	9 (8.0%)
Cladribine	4 (3.6%)
Dimethyl fumarate	16 (14.3%)
Fingolimod	3 (2.7%)
Glatiramer acetate	9 (8.0%)
Interferon beta-1a	6 (5.4%)
Natalizumab	4 (3.6%)
Ocrelizumab	6 (5.4%)
Peginterferon beta-1a	1 (0.9%)
Teriflunomide	5 (4.5%)
None	49 (43.8%)

Abbreviations: CIS: clinically isolated syndrome; RRMS: relapsing-remitting multiple sclerosis; SPMS: secondary progressive multiple sclerosis; PPMS: primary progressive multiple sclerosis; EDSS: expanded disability status scale; MS DMTs: multiple sclerosis specific disease-modifying therapies.

**Table 2:** Mean scores of measures of assessing upper extremity function and MSQOL-54.

ARAT scores mean ± SD (*n* = 111^∗^)
Both hands = 54.1 ± 6.4
Dominant hand = 54.6 ± 5.6
Nondominant hand = 53.6 ± 8.2
Less impaired hand = 55.0 ± 5.3
More impaired hand = 53.2 ± 8.3
9-HPT scores mean ± SD (*n* = 111^∗^)
Both hands = 29.1 ± 23.2
Dominant hand = 25.2 ± 8.9
Nondominant hand = 33.0 ± 41.8
Less impaired hand = 23.8 ± 6.8
More impaired hand = 34.4 ± 41.9
UEFS score mean ± SD(*n* = 112) = 22.4 ± 17.1
MSQOl-54 scores mean ± SD (*n* = 112)
PHCS = 57.7 ± 19.4; MHCS = 65.1 ± 22.1

^∗^One participant chose not to participate in the 9-HPT and ARAT but completed the UEFS and MSQOL-54. Abbreviations: ARAT: Action Research Arm Test; 9-HPT: Nine-Hole Peg Test; UEFS: Upper Extremity Function Scale; MSQOL-54: Multiple Sclerosis Quality of Life-54; PHCS: physical health composite score; MHCS: mental health composite score; SD: standard deviation.

**Table 3:** Correlations between various measures of assessing upper extremity function (ARAT/9-HPT/UEFS) and MSQOL-54 scores.

Scores	9-HPT dominant hand score	9-HPT nondominant hand score	9-HPT less impaired hand score	9-HPT more impaired hand score	9-HPT both hands score	UEFS score	PHCS	MHCS
ARAT dominant hand score	-0.403; *p* < 0.001^∗^	-0.350; *p* < 0.001^∗^	-0.379; *p* < 0.001^∗^	-0.395; *p* < 0.001^∗^	-0.410; *p* < 0.001^∗^	-0.333; *p* < 0.001^∗^	0.243; *p* = 0.010^∗^	0.072; *p* = 0.452

ARAT nondominant hand score	-0.303; *p* = 0.001^∗^	-0.396; *p* < 0.001^∗^	-0.312; *p* = 0.001^∗^	-0.400; *p* < 0.001^∗^	-0.389; *p* < 0.001^∗^	-0.349; *p* < 0.001^∗^	0.291; *p* = 0.002^∗^	0.185; *p* = 0.052

ARAT less impaired hand score	-0.255; *p* = 0.007^∗^	-0.275; *p* = 0.004^∗^	-0.258; *p* = 0.007^∗^	-0.283; *p* = 0.003^∗^	-0.285; *p* = 0.003^∗^	-0.393; *p* < 0.001^∗^	0.245; *p* = 0.010^∗^	0.146; *p* = 0.125

ARAT more impaired hand score	-0.445; *p* < 0.001^∗^	-0.473; *p* < 0.001^∗^	-0.429; *p* < 0.001^∗^	-0.512; *p* < 0.001^∗^	-0.51; *p* < 0.001^∗^	-0.31; *p* = 0.001^∗^	0.295; *p* = 0.002^∗^	0.122; *p* = 0.201

ARAT both hands score	-0.405; *p* < 0.001^∗^	-0.445; *p* < 0.001^∗^	-0.398; *p* < 0.001^∗^	-0.474; *p* < 0.001^∗^	-0.471; *p* = <0.001^∗^	-0.371; *p* < 0.001^∗^	0.327; *p* < 0.001^∗^	0.169; *p* = 0.077

UEFS score	0.355; *p* < 0.001^∗^	0.333; *p* < 0.001^∗^	0.331; *p* < 0.001^∗^	0.364; *p* < 0.001^∗^	0.354; *p* < 0.001^∗^	N/A	-0.589; *p* < 0.001^∗^	-0.406; *p* < 0.001^∗^

PHCS	-0.370; *p* < 0.001^∗^	-0.336; *p* < 0.001^∗^	-0.387; *p* < 0.001^∗^	-0.326; *p* < 0.001^∗^	-0.358; *p* < 0.001^∗^	-0.589; *p* < 0.001^∗^	N/A	0.710; *p* < 0.001^∗^

MHCS	-0.210; *p* = 0.027^∗^	-0.141; *p* = 0.139^∗^	-0.216; *p* = 0.023^∗^	-0.128; *p* = 0.180	-0.165; *p* = 0.083	-0.406; *p* < 0.001^∗^	0.710; *p* < 0.001^∗^	N/A

^∗^ *p* value is significant at *α* = 0.05. Note: negligible to low correlations were found between the scores obtained from objective measures of assessing UE function (ARAT and 9-HPT) and PHCS of MSQOL-54. Abbreviations: ARAT: Action Research Arm Test; 9-HPT: Nine-Hole Peg Test; UEFS: Upper Extremity Function Scale; MSQOL-54: Multiple Sclerosis Quality of Life-54; PHCS: physical health composite score; MHCS: mental health composite score; N/A: not applicable.

## Data Availability

The data used to support the findings of this study have not been made available because we do not have ethical permission to share or release any primary research data.

## Abbreviations

UEFS: Upper Extremity Function Scale
MSQOL-54: Multiple Sclerosis Quality of Life-54
9-HPT: Nine-Hole Peg Test
ARAT: Action Research Arm Test
*r*_*s*_: Spearman's correlation coefficient
QOL: Quality of life
UE: Upper extremity
ADL: Activities of daily living
iMS: Individuals with multiple sclerosis
PROM: Patient-reported outcome measure
EDSS: Expanded disability status scale
DMT: Disease-modifying therapy.
